# Spatial epidemiology of Japanese encephalitis virus and other infections of the central nervous system infections in Lao PDR (2003–2011): A retrospective analysis

**DOI:** 10.1371/journal.pntd.0008333

**Published:** 2020-05-26

**Authors:** Sayaphet Rattanavong, Audrey Dubot-Pérès, Mayfong Mayxay, Manivanh Vongsouvath, Sue J. Lee, Julien Cappelle, Paul N. Newton, Daniel M. Parker

**Affiliations:** 1 Lao-Oxford-Mahosot Hospital-Wellcome Trust Research Unit (LOMWRU), Microbiology Laboratory, Mahosot Hospital, Vientiane, Lao PDR; 2 Centre for Tropical Medicine and Global Health, Nuffield Department of Clinical Medicine, University of Oxford, Churchill Hospital, Oxford, United Kingdom; 3 Unité des Virus Émergents (UVE: Aix-Marseille Univ–IRD 190 –Inserm 1207 –IHU Méditerranée Infection), Marseille, France; 4 Institute of Research and Education Development, University of Health Sciences, Vientiane, Lao PDR; 5 Mahidol-Oxford Tropical Medicine Research Unit, Faculty of Tropical Medicine, Mahidol University, Thailand; 6 Epidemiology and Public Health Unit, Institut Pasteur du Cambodge, Phnom Penh, Cambodia; 7 CIRAD, UMR ASTRE, F-34398, Montpellier, France; 8 UMR ASTRE, CIRAD, INRA, Montpellier University, Montpellier, France; 9 UMR EpiA, INRA, VetAgro Sup, Marcy l’Etoile, France; 10 Department of Population Health and Disease Prevention, University of California, Irvine, United States of America; 11 Department of Epidemiology, School of Medicine, University of California, Irvine, United States of America; University of California Davis, UNITED STATES

## Abstract

**Background:**

Central nervous system (CNS) infections are important contributors to morbidity and mortality and the causative agents for ~50% patients are never identified. The causative agents of some CNS infections have distinct spatial and temporal patterns.

**Methodology/Principal findings:**

Here we present the results of a spatial epidemiological and ecological analysis of CNS infections in Lao PDR (2003–2011). The data came from hospitalizations for suspected CNS infection at Mahosot Hospital in Vientiane. Out of 1,065 patients, 450 were assigned a confirmed diagnosis. While many communities in Lao PDR are in rural and remote locations, most patients in these data came from villages along major roads. Japanese encephalitis virus ((JEV); n = 94) and *Cryptococcus* spp. (n = 70) were the most common infections. JEV infections peaked in the rainy season and JEV patients came from villages with higher surface flooding during the same month as admission. JEV infections were spatially dispersed throughout rural areas and were most common in children. *Cryptococcus* spp. infections clustered near Vientiane (an urban area) and among adults.

**Conclusions/Significance:**

The spatial and temporal patterns identified in this analysis are related to complex environmental, social, and geographic factors. For example, JEV infected patients came from locations with environmental conditions (surface water) that are suitable to support larger mosquito vector populations. Most patients in these data came from villages that are near major roads; likely the result of geographic and financial access to healthcare and also indicating that CNS diseases are underestimated in the region (especially from more remote areas). As Lao PDR is undergoing major developmental and environmental changes, the space-time distributions of the causative agents of CNS infection will also likely change. There is a major need for increased diagnostic abilities; increased access to healthcare, especially for rural populations; and for increased surveillance throughout the nation.

## Author summary

Infections of the central nervous system (CNS) are important with regard to public health. However many CNS infections are never diagnosed. In this analysis we investigated spatial and temporal patterns in hospitalized patients with suspected CNS infections in Lao PDR. We found that patients were most likely to come from villages located along major roads and highways. Patients from remote areas may have more difficulty reaching healthcare facilities. The most commonly diagnosed infection in these patients was Japanese encephalitis virus (JEV). Patients with this disease came from locations that were optimal for the mosquito vectors that spread JEV, rural areas with surface water and during the rainy season. Our analyses suggest that CNS infections should be a priority for public health workers in the region. Diagnostic capabilities should be increased throughout the nation; surveillance efforts should be broadened; and efforts should be increased toward providing easy access to healthcare for rural and remote populations.

## Introduction

Numerous illnesses go undiagnosed and the causative agents of many infections are never identified. In regions where access to healthcare facilities is limited and where diagnostic capabilities are few, a smaller proportion of diseases are objectively diagnosed. Diseases with mild symptoms may more frequently go untreated, but in some areas even severe illnesses commonly go undiagnosed and untreated. Infections of the central nervous system (CNS) can be particularly severe, affecting the brain and/or spinal cord and/or the surrounding meninges, frequently resulting in death.

Pathogens that invade and infect the CNS include viruses, bacteria, fungi, parasites, and prions. As with other infections, the causative agent(s) of many CNS infections are never determined (frequently <50%) [1–3]. Symptoms of CNS infection can range widely, even for single causative agents, leading to further difficulties with diagnosis. Diagnoses are frequently presumptive and non-specific (i.e. meningitis is often presumed to be caused by bacteria whereas encephalitis is presumed to be caused viruses [2,4–6]). Some causative agents are specific to regions (e.g. Japanese Encephalitis, Saint Louis Encephalitis, Rift Valley Fever Viruses) and exhibit seasonal fluctuations (e.g. vector borne infections), therefore geography and seasonality can facilitate presumptive diagnosis of CNS diseases [4,7].

In Southeast (SE) Asia, CNS infections are increasingly recognized as important contributors to morbidity and mortality [1,8,9]. However, detailed medical and epidemiological data are frequently lacking, especially for low income nations and from remote areas within middle-to-high income nations. Known important viral CNS infections in SE Asia include Japanese encephalitis, dengue, and rabies viruses [1]. Important bacterial CNS infections include *Streptococcus pneumoniae*, *Haemophilus influenzae*, *S*. *suis*, *Mycobacterium tuberculosis* and *Neisseria meningitidis*. *Orientia tsutsugamushi*, *Rickettsia typhi* and *Leptospira* spp. are increasingly recognized as important causes [9]. Detailed analyses that confirm the cause of CNS related infections or assess their spatial and temporal distribution in the region are rare [10–13]. Several CNS infections are vaccine preventable, however high costs of some vaccines, and geographic accessibility can limit their reach. Vaccines for JEV were first introduced in 6 provinces of Northern Lao PDR in 2011–2012. In 2013 the WHO approved a less expensive vaccine (produced in China by the Chengdu Institute of Biological Products) which has since been used in mass vaccination campaigns in Lao PDR in 2013 and 2015 [14]. Since 2015 JEV vaccine has been provided nationwide. Hib vaccine was introduced in 2009 and combined with four other vaccines (D-P-T and Hepatitis B), referred to as Pentavalent vaccines [15]. Pneumococcal vaccine was introduced in 2013 [16]. While meningococcal vaccines exist, they are not available in Lao PDR.

Recently we published the results of a study of the etiology and impact of CNS infections diagnosed among 1,065 patients at Mahosot Hospital, Vientiane, Lao PDR [17]. The goal of this secondary analysis was to investigate the spatial distribution(s) of CNS-related infections; to look for differential spatial distributions for major causative agents; and to explore potential geographic, demographic, and environmental correlates of these infections.

## Data and methods

### Data sources, processing and merging

Data used in this research were compiled from four main sources (**S1 Fig**). The epidemiological data come from an 8-year research project on CNS infections in Lao PDR from all patients who received diagnostic lumbar puncture (LP) at Mahosot Hospital in Vientiane, Lao PDR between January 2003 and August 2011 and consenting to participate [17]. All patients were admitted to the hospital because of suspected CNS infection and Mahosot Hospital is the only medical facility in Lao PDR capable of performing diagnostic LP and cerebral spinal fluid (CSF) analysis. LP was performed on all patients with suspected CNS infection, who presented with altered consciousness or neurological findings, and who were not contraindicated for LP. Tests for a large panel of pathogens were then performed at the Microbiology Laboratory following international standards (details provided in **S1 Text** and [17]). Demographic (age, gender, ethnicity) and geographic (home village) characteristics of patients were recorded.

The epidemiological data were used to create two separate datasets: One aggregated at the village level (one row per location) and another was maintained at the individual level, with one row per individual. The official Lao PDR censuses from 2005 and 2015 were used to geocode villages (based on village name and administrative units listed in patient records) and to assign village population estimates to each village (taking a mean population size between 2005 and 2015). Village location and population sizes were then merged to both the individual- and village-level datasets.

A subset of villages within the geographic region of the home villages of included patients was selected by overlaying a standard deviational ellipse with 3 standard deviations (calculations described in **S2 Text**) around the patient home villages and then selecting all villages within that ellipse (**S2 Fig**). These villages were then retained for village level comparisons between villages populated, and not populated, with patients admitted with CNS disease needing an LP. This subset was chosen to ensure that the analysis was limited to the region from which the cases for this project came, thereby using villages that share similar geographic attributes for comparison. Using all villages within the nation would have meant the inclusion of parts of Lao PDR (especially in the far south) that have very different environmental patterns. This subset of villages is hereafter referred to as the “study area”.

Major road network data was taken from OpenStreetMaps (http://www.openstreetmap.la), selecting “primary”, “secondary”, and all major connecting roads (downloaded in February 2017, **S3 Fig**). Primary and secondary roads are the two largest road classifications for the nation. Primary roads link major towns and cities and secondary roads link mid-sized towns. Primary and secondary link roads are ramps or slip roads that connect other roads to primary or secondary roads. Together, these types of roads are hereafter referred to as “major roads”. Smaller roads were not included in this analysis as they are less likely to be accurately included in the OpenStreetMaps data. The Euclidian distance was then calculated from all villages in the census to the nearest point along a major road. These distances were merged to both the village- and the individual-level datasets.

Environmental predictor variables for vegetation and surface water were derived from Moderate Resolution Imaging Spectroradiometer (MODIS) products (MOD13Q1/MYD13Q1 250 meter AQUA/TERRA 16 day composites). Since many infectious diseases, especially vector borne diseases, are strongly influenced by environmental factors we hypothesized that indicators of vegetation and surface flooding would correlate with some specific diagnoses. Three environmental indices were downloaded and considered in these analyses: a normalized flooding index (NFI) [18]; the normalized difference vegetative index (NDVI); and the enhanced vegetation index (EVI). NFI is indicative of surface water, NDVI is indicative of green surface vegetation, and EVI is an improved measure of green vegetation that is intended to account for dense forest canopies and atmospheric conditions that can lead to error in NDVI measurements. Data were downloaded for each of these environmental indices (EI) within each 16-day time period from February 2002 through December of 2011. The final analyses conducted in this research retained only the EVI and NFI for environmental predictors (summary statistics for NDVI are included) because NDVI and EVI were strongly correlated. The EIs are described in more detail in **S3 Text**.

The environmental raster data were then summarized and extracted based on varying buffer sizes (2km, 5km, and 10km) for each village in the individual- and village-level datasets. Permanent water bodies (such as the Mekong River and Nam Ngum Dam) were masked from the NFI calculations. For the village-level datasets, mean values of each environmental variable was calculated for the study period duration and used as an indicator of “average” vegetation or surface water characteristics of each village. For the individual-level dataset the values were extracted based on the admission date, using increasing durations of time prior to admission (within the same month, within the previous 2 months, within the previous year).

The final datasets include the village-level data, that is a subsample of 98% of all villages with patients included in the study and the other census designated villages within the same region (the study area), and the individual level dataset that includes all patients included in the study. Variables used in this analysis and their descriptions are listed in **Table 1**.

**Table 1:** List of variables, their spatial and temporal scales, and transformations.

Variable	description	spatial scale	temporal scale	transformation
*Village population*	Population estimate of LP patient home village. This is calculated as a mean population value from the Lao PDR official census from years 2005 and 2015.			For multivariable regressions, this variable was centered on its mean and standardized by its standard deviation.
*Distance to major road*	This is the distance in meters from the LP patient home village and the nearest point on a major highway network, from the OpenStreetMaps map layer. This distance was transformed into kilometers and rounded to the nearest 5 kilometers in order to account for measurement error.	This distance was transformed into kilometers and rounded to the nearest 5 kilometers in order to account for measurement error.		For multivariable regressions, this variable was centered on its mean and standardized by its standard deviation.
*Village elevation*	This is the elevation of the LP patient home village, calculated from a digital elevation model. It is in meters above sea level.	at village point		For multivariable regressions, this variable was centered on its mean and standardized by its standard deviation.
*NFI*	This is the normalized flooding index, described in detail in the Supporting materials.	Mean values at 2km, 5km, and 10km buffers around each village.	For village level analysis: calculated as a mean value for the study duration. For individual level analysis: Calculated for the same month (same 16 day time period); the previous 2 months (mean of the previous 5 16 day intervals); and the previous year (mean of the previous 23 16 day intervals).	Aggregated into quartiles for multivariable regressions.
*NDVI*	This is the normalized difference vegetation index, detailed in the Supporting materials.	Mean values at 2km, 5km, and 10km buffers around each village.	For village level analysis: calculated as a mean value for the study duration. For individual level analysis: Calculated for the same month (same 16 day time period); the previous 2 months (mean of the previous 5 16 day intervals); and the previous year (mean of the previous 23 16 day intervals).	
*EVI*	This is the enhanced vegetation index, detailed in the Supporting materials.	Mean values at 2km, 5km, and 10km buffers around each village.	For village level analysis: calculated as a mean value for the study duration. For individual level analysis: Calculated for the same month (same 16 day time period); the previous 2 months (mean of the previous 5 16 day intervals); and the previous year (mean of the previous 23 16 day intervals).	Aggregated into quartiles for multivariable regressions.
*Gender*	Binary for male or female, self reported in hospital records			
*Age*	Self reported age in years.			Aggregated into age groups for multivariable regressions.
*Year*	The year of admission to the hospital			For multivariable regressions, this variable was centered on its mean and standardized by its standard deviation.
*Quarter*	The calendar quarter of admission (Jan—March; Apri—June; July—Sep; Oct—Dec)			

### Exploratory spatial data analysis

Summary statistics (median; Q1:Q3; mean) were calculated for the distances between villages and the nearest major road, and aggregated by whether or not the village was home to included patients and by specific diagnoses. Summary statistics (mean and 95% confidence intervals) were also calculated for all environmental variables, at each buffer size and temporal resolution, and for each of the major diagnoses. Tukey’s post hoc range test was used to assess statistically significant differences in environmental indices across diagnoses.

Standard distance deviations (SDDs) and standard deviation ellipses (SDEs) were calculated (**details in S2 Text**) and mapped to measure and visually analyze the central tendency and spatial distributions for all patient home villages and by each of the major mono-infection diagnoses.

### Formal analyses

Multivariable regressions were used to calculate model-adjusted odds ratios and confidence intervals. The regressions at the village level focused on study patient villages and the home villages of JEV diagnosed patients. A multivariable regression was also done at the individual level focusing on JEV infected patients. Other diagnoses were not included in these analyses because of small numbers of cases per village.

Logistic generalized additive models (GAMs) were used for variable selection and specification (**detailed in S4 Text and in Table 1**) for the final models. The GAMs were used to examine different specifications of the continuous environmental, geographic (i.e. latitude and longitude), and demographic variables and for changes in model fit and strength of association across buffer sizes (i.e. 2km, 5km, or 10km buffers) and for different time durations for EI measurements (i.e. same month, 2 months prior, 12 months prior to hospital admission). The final model covariates were chosen based on a combination of *a priori* hypotheses, model fit (using the Akaike information criterion), and strength of association between the covariate and the model outcome variable. Since the home villages of patients appeared to be spatially autocorrelated, we also tested logistic regressions with the inclusion of an autocovariate term that accounted for a given village being located near other villages with cases.

The final model for the individual-level analysis was a generalized logistic mixed model with a random effect for home village. The final model for the village-level analysis was a logistic regression.

### Software

All maps were created using QGIS version 3.4.9. R cran version 3.5.2 was used for downloading, processing, and wrangling MODIS data (using the “MODIStsp”; “raster”; “rgdal”; and “maptools” packages) and for all regressions. The “mgcv” package was used for GAMs and the lme4 package was used for the generalized mixed models. Euclidian distances between villages and major roads were calculated using QGIS. ArcMap version 10.5.1 was used to calculate SDDs and SDEs.

### Ethics approval

Ethical clearance for the CNS study was granted by the Oxford University Tropical Ethics Research Committee and by the Ethical Review Committee of the Faculty of Medical Sciences, National University of Laos. Between 2003 and 2006, both Oxford Tropical Ethics Committee and the Ethical Review Committee of the Faculty of Medical Sciences, National University of Laos, approved the use of oral witnessed consent. Oral consent was confirmed by a signed witness statement. Verbal consent (from 2003–2006) and written consent (from 2006–2011) were obtained from all recruited patients or immediate relatives. All ages were included provided that they or their guardian gave witnesses oral informed consent (2003–2006) and written informed consent since 2006.

## Results

### Summary statistics

A total of 1,065 patients were recruited with no LP contraindications and consented to have a diagnostic LP; 450 (42%) were assigned a final laboratory diagnosis. The most common of these were Japanese encephalitis virus ((JEV) 94 individuals); followed by *Cryptococcus* spp. with 70 individuals (9 were *C*. *gattii*); scrub typhus (*Orientia tsutsugamushi*) 31; Dengue virus 27; *Leptospira* spp. 25; murine typhus (*Rickettsia typhi*) 24; *Streptococcus pneumoniae* in 22 and 20 with *Mycobacterium tuberculosis*. 124 patients died prior to discharge (out of 893 with recorded discharge type).

The majority (666, 63%) of patients were male, with the lowest sex bias in cryptococcal infections (40/70, 57% male) and the highest among dengue infections (22/27, 82% male) (**Table 2**). Age patterns were evident in JEV and cryptococcal infections, with median ages of 13 and 33 years, respectively (**Table 2**). Patients were linked to 582 different villages (multiple patients could come from the same village): 90 villages with JEV patients, 66 with cryptococcal patients, 31 with scrub typhus patients, 27 with dengue patients, 24 with leptospiral patients, and 24 with murine typhus patients. The majority (870, 82%) of patients came from within Vientiane Prefecture (678, 64%) or Vientiane Province (192, 18%). Vientiane City, where Mahasot Hospital is located, is in Vientiane Prefecture and Vientiane Province is adjacent to the prefecture, immediately to the North and East.

**Table 2:** Age and gender of study patients. (Q1 and Q3 indicate the first and third quartiles, respectively).

	male/female	M/F ratio	median age in years (Q1 –Q3)	Total number
**all patients**	666/399	1.67	23 (8–38)	1065
**JEV**	55/39	1.41	13 (8–20)	94
***Cryptococcus* spp.**	40/30	1.33	33 (27–41)	70
**scrub typhus**	22/9	2.44	16 (9–29)	31
**Dengue virus**	22/5	4.4	20 (9–30)	27
***Leptospirosis* spp.**	17/8	2.13	25 (12–39)	25
**murine typhus**	17/7	2.43	32 (16–51)	24

A total of 6,416 villages (of 10,520 recorded in 2005 [19]) were selected as the study area for further village level analyses (**Table 3**). Villages that were home to study patients were 11 times (0.7km versus 6.3km, from **Table 3**) closer to a major road when compared to other villages within the study area. Scrub typhus and JEV infected patient homes were further from major roads than other patients, but the difference was not statistically significant in univariate analyses.

**Table 3:** Distribution of distances (in km) to the nearest major road, by diagnosis type. Counts of villages are from within 3 standard deviational ellipses (SDEs) of all LP villages (referred to as the “study area” in text). In some cases, multiple patients came from the same village meaning that counts of villages will be smaller than counts of total patients. (Q1 and Q3 indicate the first and third quartiles, respectively).

	n	median distance in km (Q1—Q3)
**All**	6416	5.4 (0.5–15.2)
**Villages without study patient**	5847	6.3 (0.8–16.1)
**Villages with study patient**	569	0.7 (0.1–4.1)
**JEV**	88	0.6 (0.1–8.0)
***Cryptococcus* spp.**	66	0.3 (0.1–1.4)
**scrub typhus**	31	0.6 (0.2–3.5)
**Dengue virus**	27	0.3 (0.1–1.1)
***Leptospirosis* spp.**	22	0.4 (0.1–1.9)
**murine typhus**	24	0.3 (0.1–2.2)

**Fig 1:**
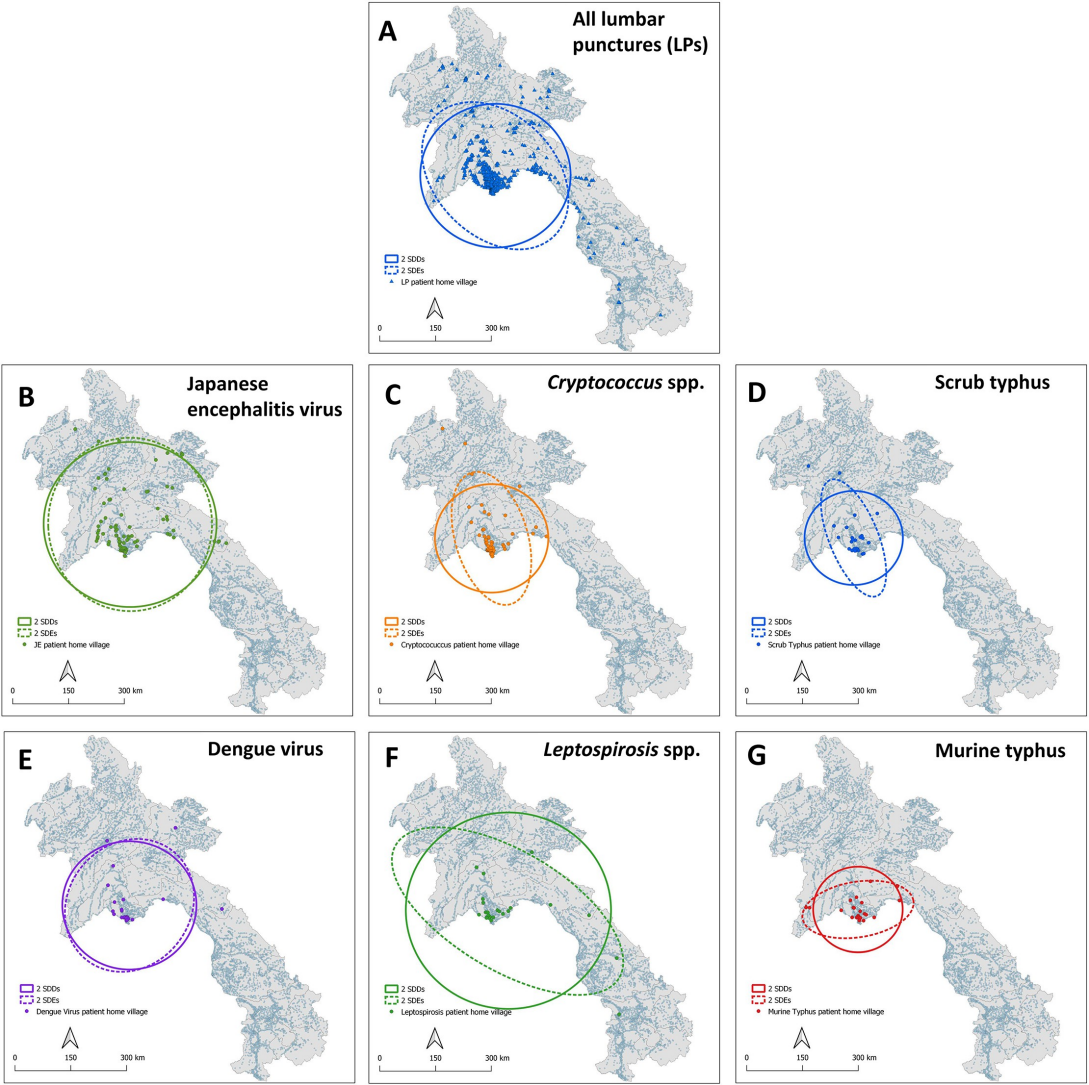
Spatial distributions of the home villages of study patients, for A: all study patients, B: study patients with JEV infections, and C: with cryptococcal infections, D: scrub typhus infections, E: with dengue virus infections, F: with leptospiral infections, and G: with murine typhus infections. SDDs and SDEs are weighted by case numbers, with some patients coming from the same village. Maps were created using QGIS version 3.4.9. All layers were created by the authors of this manuscript.

The home villages of JEV patients were more broadly dispersed (**Fig 1B**) than for patients with other etiologies (**Fig 1C**), evident from the circular, larger SDE and SDD. The distribution of these JEV patient home villages was also relatively isotropic, with the SDE and SDD being nearly identical. Conversely, patients with cryptococcal infections were clustered near Vientiane City and along the road leading North/Northwest from the urban center (**Fig 1C**). Scrub typhus and murine typhus infections were also both clustered around Vientiane City but showed perpendicular spatial distributions (**S4 Fig and S5 Fig**) a pattern previously described from IgG seropositivity data from Vientiane City [20]).

### Characteristics of patient home villages

**Fig 2:**
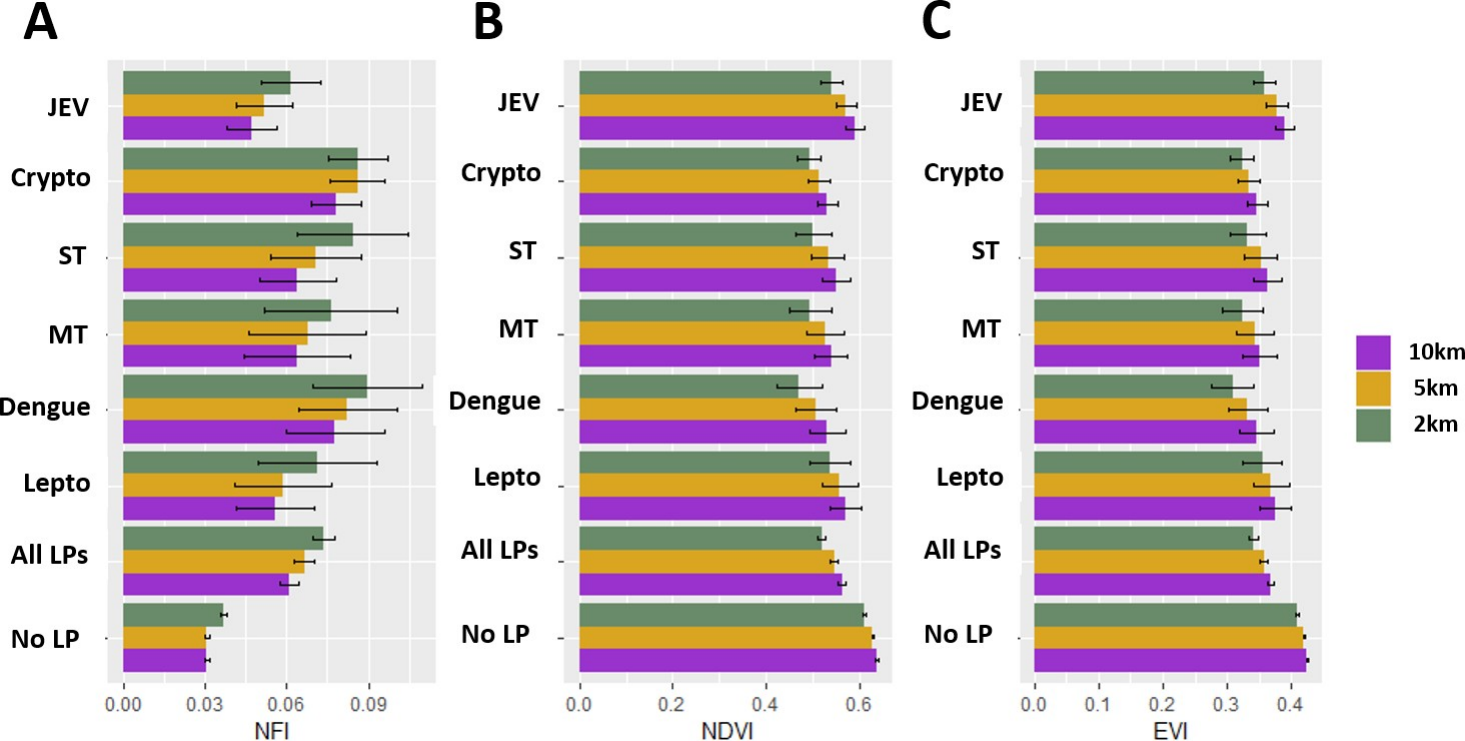
Environmental indices for villages with study patient homes for the duration of the study period (January 2003 through August 2011) for all study patient villages, non study patient villages in the study area, and for major diagnoses (JEV = Japanese Encephalitis virus; Crypto = cryptococcal infection; ST = scrub typhus; MT = murine typhus; dengue = Dengue virus; lepto = *Leptospira* spp. infection). Bar values are mean values and the error bars are 95% confidence intervals, using the t-distribution. NFI values here have a constant (0.25) added to them for visualization only.

**Fig 3:**
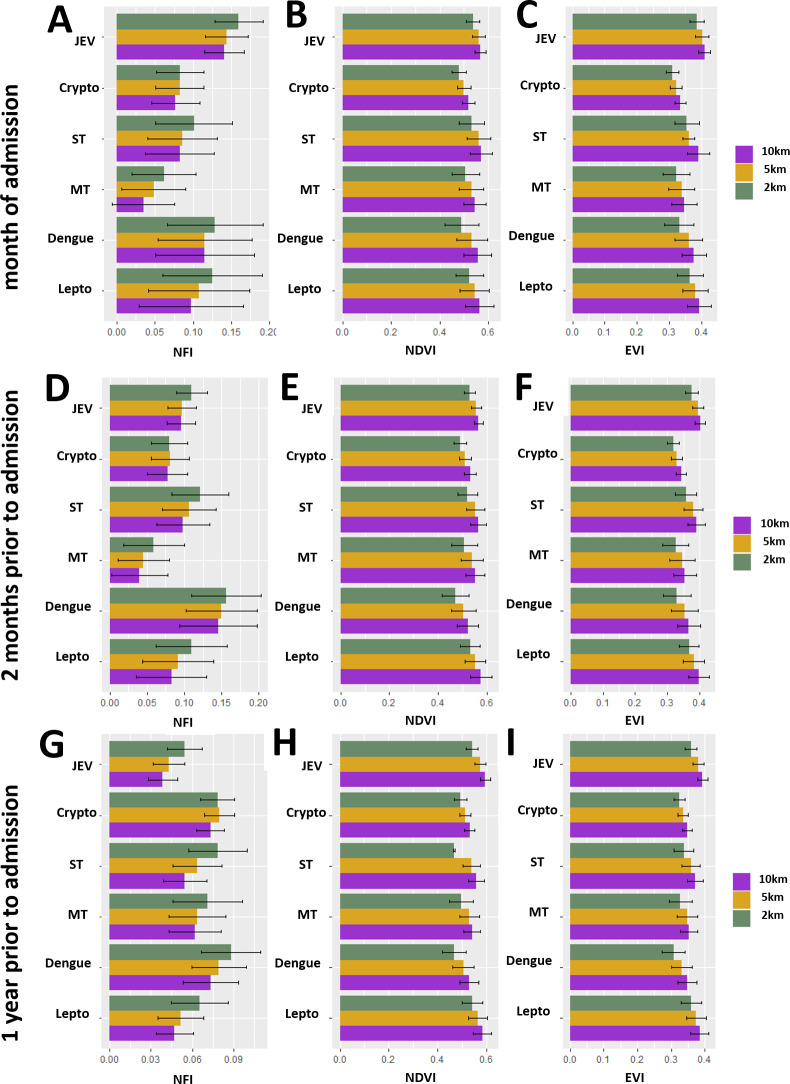
Environmental indices for study patients by major diagnosis and at different times leading up to the date of admission. JEV = Japanese Encephalitis virus; Crypto = cryptococcal infection; ST = scrub typhus; MT = murine typhus; dengue = Dengue virus; lepto = *Leptospira* spp. infection. Bar values are mean values and the error bars are 95% confidence intervals, using the t-distribution. NFI values here have a constant (0.25) added to them for visualization only.

Mean NFI values for the 2km radius tended to be higher than for either the 5km or 10km radius as surface flooding is heterogeneous and taking a mean across larger radii dilutes the measurement. The opposite pattern was observed for both vegetation indices. The 2km radius for both mean NDVI and mean EVI was usually smaller than at 5km or 10km radii (**Figs 2 and 3**).

Study patient villages had higher mean NFI values than non-study patient villages (**Fig 2A**) (non-study patient villages are those in the same study area as study patient villages but were not home to a study patient). The home villages of study patients diagnosed with dengue virus and cryptococcal infections had high mean NFI values over the duration of the study period when compared to other major diagnoses (**Fig 2A**).

Conversely, the home villages of study patients tended to have lower mean EVI values than non-study patient villages (**Fig 2C**). Villages from which patients who were diagnosed with JEV were an exception to this general pattern. JEV patient home villages had higher mean EVI when compared to home villages of patients with dengue and cryptococcal infections (**Fig 2C**).

Home villages of patients with JEV diagnoses had lower mean NFI over the duration of the study period, but had higher NFI than other major diagnoses when looking specifically at the month of admission (especially when compared to cryptococcal infections and murine typhus (**Fig 3A**)). JEV patient home villages also had higher EVI during the month of admission than most other mono-infections, especially when compared to either cryptococcal infections or murine typhus (**Fig 3C**). Scrub typhus infections had higher EVI than cryptococcal infections when the measurement was taken at the 10km radius buffer (not detectable at smaller radii (**Fig 3C**)).

Home villages of patients with dengue infections had higher NFI than murine typhus or *Cryptococcus* spp. patient home villages when considering the 2 months prior to admission (**Fig 3D**). *Cryptococcus* spp. patient home villages had particularly low EVI in the two months leading up to admission, especially when compared to JEV and *Leptospira* spp. patient home villages (**Fig 3F**).

At one year prior to admission both *Cryptococcus* spp. patient home and dengue virus patient home villages had higher NFI than JEV patient home villages (**Fig 3G**).

### Logistic regressions for geographic, environmental and demographic predictors of CNS infections

#### Village level regression with dichotomous outcome variable for whether or not a village had a case

In agreement with univariate analyses, villages from which study patients came tended to be larger in population size (**S4 Fig**), lower in elevation (**S5 Fig**), and closer to a major road when compared to other villages within the study area (**Table 4**). They also had higher mean levels of surface flooding, with villages in the highest NFI quadrant having over two times the odds (AOR: 2.21; CI: 1.49–3.31) of being a home village for study patients when compared to neighboring villages in the study area (**Table 4 and Fig 2**).

**Table 4:** Logistic regression and model adjusted odds ratios (AOR) for village level analysis of LP villages. Villages in which a CNS infection was diagnosed were compared to villages with no diagnosed infections.

covariate	total	LP count	AOR (CI)
NDFI Q1	1604	68	reference group
NDFI Q2	1604	75	1.07 (0.73–1.56)
NDFI Q3	1604	130	**1.48 (1.03–2.16)**
NDFI Q4	1604	296	**2.21 (1.49–3.31)**
EVI Q1	1604	311	reference group
EVI Q2	1604	134	1.16 (0.86–1.57)
EVI Q3	1604	74	1.12 (0.76–1.66)
EVI Q4	1604	50	1.19 (0.74–1.91)
Village population			**2.22 (2.03–2.42)**
Elevation			**0.52 (0.42–0.63)**
Distance to major road			**0.68 (0.57–0.80)**

#### Village level regression with dichotomous outcome variable for whether or not a village had a JEV case

Villages from which JEV patients originated had few defining characteristics in the logistic regression, other than being larger in population size (AOR: 1.74; CI: 1.55–1.96) and at lower elevations (AOR: 0.69; CI: 0.46–0.97) than non-study patient villages (**Table 5**).

**Table 5:** Logistic regression and model adjusted odds ratios (AOR) for village level analysis of JEV villages. Villages in which a JEV infection occurred are compared to villages with no diagnosed CNS infections.

covariate	total	JEV count	AOR (CI)
NDFI Q1	1604	18	reference group
NDFI Q2	1604	15	0.83 (0.38–1.76)
NDFI Q3	1604	20	1.11 (0.53–2.35)
NDFI Q4	1604	35	1.26 (0.54–2.95)
EVI Q1	1604	35	reference group
EVI Q2	1604	23	1.81 (0.87–3.76)
EVI Q3	1604	17	1.85 (0.77–4.46)
EVI Q4	1604	13	1.76 (0.63–4.93)
Village population			**1.74 (1.55–1.96)**
Elevation			**0.69 (0.46–0.97)**
Distance to major road			0.88 (0.62–1.20)

#### Individual level regression with dichotomous outcome variable for whether or not an individual had a JEV infection

In the individual-level analysis (**Table 6**), age and season were the strongest predictors of JEV infections among all patients. Patients who were admitted between July and September had over seven times the odds (AOR: 7.40; CI: 1.45–37.67) of being diagnosed with JEV when compared to patients who were admitted between January and March (**Table 6**). JEV was most common in children aged 5 through 14 (AOR: 2.74; CI: 1.31–5.69; ages 0–4 as the comparison group). NFI during the month of admission (10km buffer used in the regression) was also a strong predictor of JEV infection. Individuals who came from villages in the highest quadrant of NFI measurements had approximately 3 times the odds being diagnosed with JEV (AOR: 3.06; CI: 1.04–8.96). While study patients came from villages with lower mean elevations, patients who were diagnosed with JEV came from higher elevation villages in comparison to the other patients (AOR: 1.36; CI: 1.11–1.66). EVI was a significant predictor in models that did not include distance to road, village population, and elevation (**Table 6 M1 and M2**).

**Table 6:** Mixed effects logistic regression and model adjusted odds ratios (AOR) for individual level analysis. Individuals with JEV infections were compared to individuals with other CNS infections.

			M1	M2	M3
covariate	total	JEV count	AOR (CI)	AOR (CI)	AOR (CI)
NDFI Q1	262	7	reference group	reference group	reference group
NDFI Q2	261	16	**2.91 (1.06–7.97)**	2.28 (0.86–6.01)	2.32 (0.87–6.19)
NDFI Q3	261	31	**2.73 (1.02–7.28)**	**2.75 (1.07–7.07)**	**2.98 (1.13–7.85)**
NDFI Q4	262	38	**3.41 (1.17–9.94)**	**3.12 (1.09–8.90)**	**3.06 (1.04–8.96)**
EVI Q1	262	8	reference group	reference group	reference group
EVI Q2	261	20	2.11 (0.84–5.28)	1.89 (0.76–4.74)	1.60 (0.63–4.04)
EVI Q3	261	24	2.04 (0.81–5.12)	1.65 (0.67–4.07)	1.33 (0.53–3.32)
EVI Q4	262	40	**4.19 (1.62–10.87)**	**3.44 (1.35–8.73)**	2.43 (0.91–6.46)
Jan—March	210	2	reference group	reference group	reference group
April—June	267	22	**5.12 (1.10–23.89)**	4.45 (0.95–20.76)	**5.05 (1.07–23.73)**
July—Sep	333	62	**8.72 (1.75–43.46)**	**6.35 (1.26–31.89)**	**7.40 (1.45–37.67)**
Oct—Dec	253	8	1.80 (0.35–9.25)	1.51 (0.29–7.81)	1.54 (0.29–8.17)
Year			**1.31 (1.03–1.68)**	**1.34 (1.05–1.72)**	1.27 (0.98–1.64)
0 through 4	208	13		reference group	reference group
5 through 14	150	37		**3.00 (1.46–6.18)**	**2.74 (1.31–5.69)**
15 through 24	192	28		1.74 (0.84–3.62)	1.37 (0.64–2.94)
25 through 34	186	10		0.66 (0.27–1.61)	0.61 (0.25–1.48)
35 through 44	133	4		**0.26 (0.07–0.96)**	**0.26 (0.07–0.97)**
45 plus	196	2		**0.11 (0.02–0.52)**	**0.10 (0.02–0.47)**
female	399	39		reference group	reference group
male	666	55		0.97 (0.60–1.58)	1.08 (0.66–1.79)
Village population					1.00 (0.77–1.29)
Elevation					**1.36 (1.11–1.66)**
Distance to major road					1.08 (0.88–1.32)

## Discussion

Patients from this study were recruited based on symptomology and a medical procedure that is only available at a single location in the nation (diagnostic LP at Mahosot Hospital, Vientiane). The home villages of all included study patients, regardless of diagnosis, were approximately centered on Vientiane City and were closer to major roads than would be expected by chance alone. For many of the infections studied in this analysis, this association is likely more related to geographic and financial access to healthcare systems rather than exposure to environmental risk factors–especially for infections that are more associated with rural areas (e.g. JEV). This finding also suggests that the results here may be a vast underestimate of the true burden of CNS infections, with much of the Lao population not being in near proximity to a major road (**Table 3**). The causative agents of CNS infections differ in biology, ecology, and geography, and this is evident through the spatial distributions of the home villages of patients. The geographic, environmental, and demographic patterns exhibited by patients needing a diagnostic LP for suspected CNS infections, and for specific diagnoses, are the result of complex overlapping factors.

A similar spatial pattern was described from an epidemiological analysis of CNS infections among children admitted to Ho Chi Minh City hospitals in Vietnam–with most patients coming from districts near the hospital [21]. While the majority of infections (55%) in the Vietnam study were presumed to be bacterial in origin, in this study from Lao PDR bacterial infections were identified in only 38% (170 out of 450 patients with diagnoses).

JEV was the single largest identified cause of CNS infections in these data; it primarily affected children (median 13 years of age, **Table 2**), occurred predominantly during the rainy season (likely corresponding to peaks in mosquito vector populations), and in villages with recent high levels of surface water [22]. JEV is a vaccine-preventable disease, but the vaccine has historically been expensive and vaccine programs are frequently limited by access to remote communities. While the vaccine is now routinely given to all children less than 9 months of age, coverage may be low in some areas.

The second largest contributor to CNS diseases were cryptococcal infections, which are opportunistic fungal infections with high mortality [23]. Of the 70 patients with cryptococcal infections, 12 died prior to discharge and another 8 likely died at home after leaving the hospital. Cryptococcal infections are generally acquired after inhalation of the yeast-like form of the fungus which has been associated with several ecological habitats (*Cryptococcus gattii* has been associated with over 50 species of trees; *Cryptococcus neoformans* has been associated with bird droppings but is also suspected to be associated with plants [24,25]). This disease has a long incubation period [26,27] and while *C*. *gattii* infections commonly occur among immunocompetent individuals, *C*. *neoformans* infections are frequently associated with HIV infections [28,29]. In these data, 20 of the patients diagnosed with cryptococcal infections also had HIV infections. While several studies have shown that cryptococcal species exist in specific ecological habitats and have inferred environmental exposure, the long incubation period and complex natural history likely obfuscate ecological correlations.

There are several limitations to this research. Diagnostic LPs are only conducted in Mahosot Hospital in the national capital. Logistical and financial difficulties in accessing healthcare facilities, and especially for etiological diagnostic capabilities, likely leads to severe under-reporting of meningitis, encephalitis, or in the diagnosing the causative agent in these conditions when the patient does access care. All of these factors ultimately lead to small case counts for numerous different causative agents. We looked for potential associations between some measures of disease severity (esp. mortality), duration of illness (e.g. days of fever) and distance to a major road. However, no straightforward patterns were evident. The spatial patterns in points (villages) and ellipses exhibited in these data are likely influenced by the shape of the nation and it is possible that the point patterns and ellipses would differ if we had data from neighboring nations. Spatial and temporal patterns that differentiate different infections might be more obvious if the surveillance system instead focused on any symptomatic infections (rather than only suspected infections of the CNS). Some pathogens are neurotropic whereas others have tropism for other organs, while being capable of occasionally infecting the CNS. This may partially explain the higher case counts of JEV and why we were able to identify spatial, temporal, and environmental predictors for this causative agent.

OpenStreetMaps data are volunteered data and may be prone to error. For this reason we focused on major roads, whose routes have changed very little over the last decades. For the regressions, the distances from all villages to the nearest major road was also rounded to the nearest 5km. Examination of satellite imagery in comparison with the major roads from OpenStreetMaps suggests that where error does exist, it is on a scale of ±100 meters, meaning that measurements of distances, as used in this analysis, should not be strongly influenced. Some of these data now come from over a decade ago. Surveillance systems of this type (based on relatively vague symptomology), with a wide panel of possible contributing causative agents, and necessary intensive laboratory components are extremely labor and time intensive.

Lao PDR is currently undergoing vast environmental, demographic, and economic changes. Road networks are increasing in range and density and several areas (i.e. Vientiane, Savannakhet) are undergoing expansive urbanization [30]. These environmental changes will most likely result in shifting patterns of infectious diseases. As the region undergoes urbanization (including both an increase in urban landscape and movement of human populations to urban centers), pathogens that thrive in rural areas (e.g. JEV) may undergo reduced transmission, especially if vaccine campaigns are more capable of reaching rural populations. Conversely, infections that cluster in urban and peri-urban areas (such as dengue and murine typhus) may increase in frequency.

Several environmental indices from remote sensing instruments have shown potential for predicting disease risk, differentiating disease types, or for other surveillance efforts in SE Asia [31,32] and globally [33–37]. This analysis, and others like it, illustrates the ability to differentiate some infections (namely JEV when compared to other diagnoses) through the use of freely available data (i.e. MODIS) and software (R and QGIS) and routinely collected healthcare data. Surveillance systems and potentially diagnostic algorithms [38] in developing settings could benefit from inclusion of such resources. More comprehensive studies that investigate these important causative agents of CNS infections would provide a better understanding of the environmental drivers of these diseases. A far-reaching surveillance system that is representative of the entire nation and includes likely CNS infections would be beneficial in order to assess the true burden of CNS infections–many of which would benefit from primary and secondary prevention through increased provision of vaccines, vector control, and early diagnosis and treatment. Given the inherent difficulties in accurately diagnosing and treating CNS infections, the predictors reported here and from other epidemiological studies for major contributors to CNS diseases (i.e. age, seasonality, location, and environmental characteristics) could be considered alongside clinical symptomology when presumptive diagnoses are being made. However, it will be important to consider current and ongoing demographic, environmental, and economic changes in Lao PDR.

Finally, increasing population access to vaccines, diagnosis, and treatment would have clear benefits to overall population health. As with other parts of the developing world, a large fraction of the Lao population must travel long distances in order to reach primary healthcare centers. In 2005 73% of the Lao population was reported to live in rural areas, 21% without roads. By 2015 67% of the population were reported to live in rural villages with 8% in villages without roads [39]. For many communities, travel during the wet season remains difficult. Travel costs can also be prohibitive. Most of the CNS infections in this analysis occurred or developed symptoms during the wet season. Public health initiatives that help to decrease the distances between communities and the healthcare services that they need are warranted.

## Supporting information

S1 Text Detailed description of the diagnosis of major infectious agents. (DOCX)

S2 Text Detailed description of the analysis of spatial point patterns. (DOCX)

S3 Text Detailed description of the calculation of the Environmental Indices (EIs) used in this analysis. (DOCX)

S4 Text Statistical model selection. (DOCX)

S1 Fig Diagram of data processing and aggregation. Four different data sources are used (indicated by boxes with dashed lines). Two main datasets are created from the combined sources (indicated by boxes shaded in grey): an individual-level dataset (one row per patient) and a village-level dataset (one row per village). NDVI indicates the normalized differential vegetation index, EVI indicates the enhanced vegetation index, and NFI indicates the normalized flooding index.
(DOCX)

S2 Fig Subset of villages selected for village level analysis. A standard deviational ellipse ((SDE) with 3 standard deviations) was drawn around the home villages of all LP patients. All villages within that SDE were selected for the village level analysis.
(DOCX)

S3 Fig Major roads (dark black lines) downloaded from OpenStreetMaps (2017) for use in calculating the Euclidian distance from each village to the nearest major road. Roads included “primary”, “secondary”, and all major connecting roads.
(DOCX)

S4 Fig Mean village population (and 95% CI) by diagnosis. (DOCX)

S5 Fig Mean village elevation (and 95% CI) by LP diagnosis. (DOCX)

## References

[1] Tarantola A, Goutard F, Newton P, de Lamballerie X, Lortholary O, Cappelle J, et al Estimating the Burden of Japanese Encephalitis Virus and Other Encephalitides in Countries of the Mekong Region. PLoS Neglected Tropical Diseases. 2014;8: 4 10.1371/journal.pntd.0002533 24498443 PMC3907313

[2] Glaser C a, Honarmand S, Anderson LJ, Schnurr DP, Forghani B, Cossen CK, et al Beyond viruses: clinical profiles and etiologies associated with encephalitis. Clinical infectious diseases: an official publication of the Infectious Diseases Society of America. 2006;43: 1565–1577. 10.1086/509330 17109290

[3] Glaser CA, Gilliam S, Schnurr D, Forghani B, Honarmand S, Khetsuriani N, et al In Search of Encephalitis Etiologies: Diagnostic Challenges in the California Encephalitis Project, 1998–2000. Clinical Infectious Diseases. 2003;36: 731–742. 10.1086/367841 12627357

[4] Solomon T, Michael BD, Smith PE, Sanderson F, Davies NWS, Hart IJ, et al Management of suspected viral encephalitis in adults–Association of British Neurologists and British Infection Association National Guidelines. Journal of Infection. 2012;64: 347–373. 10.1016/j.jinf.2011.11.014 22120595

[5] McGill F, Heyderman RS, Michael BD, Defres S, Beeching NJ, Borrow R, et al The UK joint specialist societies guideline on the diagnosis and management of acute meningitis and meningococcal sepsis in immunocompetent adults. Journal of Infection. 2016;72: 405–438. 10.1016/j.jinf.2016.01.007 26845731

[6] Bharucha T, Vickers S, Ming D, Lee SJ, Dubot-Pérès A, de Lamballerie X, et al Association between reported aetiology of central nervous system infections and the speciality of study investigators—a bias compartmental syndrome? Trans R Soc Trop Med Hyg. 2017;111: 579–583. 10.1093/trstmh/try008 29474737 PMC6018877

[7] Kennedy PGE. Viral Encephalitis: Causes, Differential Diagnosis, and Management. Journal of Neurology, Neurosurgery & Psychiatry. 2004;75: i10–i15. 10.1136/jnnp.2003.034280 14978145 PMC1765650

[8] Olsen SJ, Campbell AP, Supawat K, Liamsuwan S, Chotpitayasunondh T, Laptikulthum S, et al Infectious Causes of Encephalitis and Meningoencephalitis. 2015;21: 2003–2005. 10.3201/eid2102.140291 PMC4313633 25627940

[9] Dittrich S, Rattanavong S, Lee SJ, Panyanivong P, Craig SB, Tulsiani SM, et al Orientia, rickettsia, and leptospira pathogens as causes of CNS infections in Laos: A prospective study. The Lancet Global Health. 2015;3: e104–e112. 10.1016/S2214-109X(14)70289-X 25617190 PMC4547322

[10] Mai NTH, Phu NH, Nhu LNT, Hong NTT, Hanh NHH, Nguyet LA, et al Central Nervous System Infection Diagnosis by Next-Generation Sequencing: A Glimpse Into the Future? Open Forum Infect Dis. 2017;4 10.1093/ofid/ofx046 28480297 PMC5411956

[11] Turner P, Suy K, Tan LV, Sar P, Miliya T, Hong NTT, et al The aetiologies of central nervous system infections in hospitalised Cambodian children. BMC Infect Dis. 2017;17 10.1186/s12879-017-2915-6 29284418 PMC5747189

[12] Ho NT, Hoang VMT, Le NNT, Nguyen DT, Tran A, Kaki D, et al A spatial and temporal analysis of paediatric central nervous system infections from 2005 to 2015 in Ho Chi Minh City, Vietnam. Epidemiology and Infection. 2017;145: 3307–3317. 10.1017/S095026881700228X 29061204 PMC9148745

[13] Mawuntu AHP, Bernadus JBB, Dhenni R, Wiyatno A, Anggreani R, Feliana, et al Detection of central nervous system viral infections in adults in Manado, North Sulawesi, Indonesia. PLOS ONE. 2018;13: e0207440 10.1371/journal.pone.0207440 30444898 PMC6239303

[14] UNICEF. Japanese Encephalitis Vaccine Market and Supply Update. 2015.

[15] Evdokimov K, Sayasinh K, Nouanthong P, Vilivong K, Samountry B, Phonekeo D, et al Low and disparate seroprotection after pentavalent childhood vaccination in the Lao People’s Democratic Republic: a cross-sectional study. Clinical Microbiology and Infection. 2017;23: 197–202. 10.1016/j.cmi.2016.10.007 27756713

[16] Lao PDR first S.E. Asian nation to introduce pneumococcal vaccine and demonstrates cervical cancer vaccine with GAVI support. In: WHO [Internet]. [cited 19 Feb 2020]. Available: https://www.who.int/immunization/newsroom/lao_introduction_pneumococcal_vaccine_and_cervical_cancer/en/

[17] Dubot-Pérès A, Mayxay M, Phetsouvanh R, Lee SJ, Rattanavong S, Vongsouvath M, et al Management of Central Nervous System Infections, Vientiane, Laos, 2003–2011—Volume 25, Number 5—5 2019—Emerging Infectious Diseases journal—CDC. [cited 26 Jul 2019]. 10.3201/eid2505.180914 31002063 PMC6478220

[18] Boschetti M, Nutini F, Manfron G, Brivio PA, Nelson A. Comparative analysis of normalised difference spectral indices derived from MODIS for detecting surface water in flooded rice cropping systems. PLoS ONE. 2014;9 10.1371/journal.pone.0088741 24586381 PMC3930609

[19] Lao Population and Housing Census 2015: Provisional Report. Ministry of Planning and Investment Lao Statistics Bureau; 2015 Dec p. 64. Available: https://lao.unfpa.org/sites/default/files/pub-pdf/Final%20report-editting-English1.pdf

[20] Vallé J, Thaojaikong T, Moore CE, Phetsouvanh R, Richards AL, Souris M, et al Contrasting spatial distribution and risk factors for past infection with scrub typhus and murine typhus in Vientiane city, Lao PDR. PLoS Neglected Tropical Diseases. 2010;4: 1–10. 10.1371/journal.pntd.0000909 21151880 PMC2998433

[21] HO NT, HOANG VMT, LE NNT, NGUYEN DT, TRAN A, KAKI D, et al A spatial and temporal analysis of paediatric central nervous system infections from 2005 to 2015 in Ho Chi Minh City, Vietnam. Epidemiology and Infection. 2017;145: 3307–3317. 10.1017/S095026881700228X 29061204 PMC9148745

[22] Campbell G, Hills S, Fischer M, Jacobson J, Hoke C, Hombach J, et al Estimated global incidence of Japanese encephalitis: Bulletin of the World Health Organization. 2011;89: 766–774. 10.2471/BLT.10.085233 22084515 PMC3209971

[23] Slavin MA, Chakrabarti A. Opportunistic fungal infections in the Asia-Pacific region. Medical Mycology. 2012;50: 18–25. 10.3109/13693786.2011.602989 21905945

[24] Springer DJ, Chaturvedi V. Projecting global occurrence of Cryptococcus gattii. Emerging Infectious Diseases. 2010;16: 14–20. 10.3201/eid1601.090369 20031037 PMC2874352

[25] Ellis DH, Pfeiffer TJ. Ecology, life cycle, and infectious propagule of Cryptococcus neoformans. The Lancet. 1990;336: 923–925. 10.1016/0140-6736(90)92283-N 1976940

[26] Castrodale LJ, Gerlach RF, Preziosi DE, Frederickson P, Lockhart SR. Prolonged incubation period for cryptococcus gattii infection in cat, Alaska, USA. Emerging Infectious Diseases. 2013;19: 1034–1035. 10.3201/eid1906.130006 23735429 PMC3713837

[27] Georgi A, Schneemann M, Tintelnot K, Calligaris-Maibach RC, Meyer S, Weber R, et al Cryptococcus gattii meningoencephalitis in an immunocompetent person 13 months after exposure. Infection. 2009;37: 370–373. 10.1007/s15010-008-8211-z 19390780

[28] Wright P, Inverarity D. Human immunodeficiency virus (HIV) related cryptococcol meningitis in rural Central Thailand-Treatment difficulties and prevention strategies. Southeast Asian J Trop Med Public Health. 2007;38: 4. 17539247

[29] Inverarity D, Bradshaw Q, Wright P, Grant A. The spectrum of HIV-related disease in rural Central Thailand. Southeast Asian J Trop Med Public Health. 2002;33: 10. 12757233

[30] Bank AD. Urban development in the Greater Mekong Subregion. Steinberg F, Hakim J, editors. Mandaluyong City, Philippines: Asian Development Bank; 2016 10.1111/j.1467-9787.2010.00709.x

[31] Ledien J, Sorn S, Hem S, Huy R, Buchy P, Tarantola A, et al Assessing the performance of remotely-sensed flooding indicators and their potential contribution to early warning for leptospirosis in Cambodia. Schumann GJ-P, editor. PLOS ONE. 2017;12: e0181044 10.1371/journal.pone.0181044 28704461 PMC5509259

[32] Chadsuthi S, Chalvet-Monfray K, Wiratsudakul A, Suwancharoen D, Cappelle J. A remotely sensed flooding indicator associated with cattle and buffalo leptospirosis cases in Thailand 2011–2013. BMC Infectious Diseases. 2018;18 10.1186/s12879-018-3537-3 30497412 PMC6267035

[33] Rogers DJ, Randolph SE, Snow RW, Hay SI. Satellite imagery in the study and forecast of malaria. Nature. 2002;415: 710–715. 10.1038/415710a 11832960 PMC3160466

[34] Lambin EF, Tran A, Vanwambeke SO, Linard C, Soti V. Pathogenic landscapes: Interactions between land, people, disease vectors, and their animal hosts. International Journal of Health Geographics. 2010;9: 54 10.1186/1476-072X-9-54 20979609 PMC2984574

[35] Beck LR, Lobitz BM, Wood BL. Remote sensing and human health: new sensors and new opportunities. Emerg Infect Dis. 2000;6: 217–227. 10827111 10.3201/eid0603.000301 PMC2640871

[36] Ford TE, Colwell RR, Rose JB, Morse SS, Rogers DJ, Yates TL. Using Satellite Images of Environmental Changes to Predict Infectious Disease Outbreaks. Emerg Infect Dis. 2009;15: 1341–1346. 10.3201/eid/1509.081334 19788799 PMC2819876

[37] Lleo MM, Lafaye M, Guell A. Application of space technologies to the surveillance and modelling of waterborne diseases. Curr Opin Biotechnol. 2008;19: 307–312. 10.1016/j.copbio.2008.04.001 18514503

[38] Pokharel S, White LJ, Aguas R, Celhay O, Pellé KG, Dittrich S. Algorithm in the diagnosis of febrile illness using pathogen-specific rapid diagnostic tests. Clin Infect Dis. [cited 2 9 2019]. 10.1093/cid/ciz665 31313805 PMC7245147

[39] Results of the Population and Housing Census 2015. Lao Statistics Bureau; 2016 p. 282. Available: https://lao.unfpa.org/sites/default/files/pub-pdf/PHC-ENG-FNAL-WEB_0.pdf

