# Supplementary material for: Spatial epidemiology of Japanese encephalitis virus and other infections of the central nervous system infections in Lao PDR (2003–2011): A retrospective analysis
Source: PLoS Negl Trop Dis. 2020 May 26;14(5):e0008333. doi: 10.1371/journal.pntd.0008333 (PMC7274481; doi:10.1371/journal.pntd.0008333)
Supplement: S1 Text — (DOCX) [file pntd.0008333.s001.docx]

**S1 Text: Diagnosis of major infectious agents**

A detailed report of the data collection and primary analysis can be found in [1]. Cerebrospinal fluid (CSF) was taken from all patients consenting to be included in this study (approximately 2.5 mL for children < 1yo; 3.5 mL for children 1 – 14yo; and 8 mL for patients > 15yo). A venous blood sample was also taken on the same day as the lumbar puncture (approximately 5.5 mL for patients > 15yo; 10 mL for children 1 – 14yo; and 18.5 mL for patients > 15yo). When possible follow-up serum samples were collected between 7 and 10 days post LP. All patient samples were analyzed using a panel of tests, including complete blood count; culture; biochemistry panel; and both serological and molecular assays for a range of fungi, parasites, viruses, and bacteria.

We considered sample size, natural history, and ecology of infections for selecting pathogens for this secondary analysis. Detections of the pathogens included in this analysis are as follows:

- *Japanese encephalitis virus* (JEV) infections were detected using ELISA IgM (Japanese Encephalitis/Dengue IgM Combo ELISA from Panbio) in CSF, and in serum at both admission and follow-up. Patients who were negative at admission but seropositive in a follow-up were classified as confirmed JEV infections. Some JEV infections were also diagnosed by culture or PCR.
- *Cryptococcus* spp. infections were detected using Indian ink stain of CSF; *Cryptococcus* Antigen Latex Agglutination Test with CSF (when HIV infection was suspected); and culture on Sabouraud agar when Indian ink test was positive or HIV infection was suspected.
- *Dengue virus* infections were detected using Hydrolysis probe real time RT-PCR [2] in CSF and serum; NS1 ELISA (Dengue Early ELISA from Panbio) in CSF and serum; and ELISA IgM (Japanese Encephalitis/Dengue IgM Combo ELISA from Panbio) in CSF, and in serum at both admission and follow-up (if negative at admission but seropositive in a follow-up).
- *Flavivirus* infections were detected using nested SYBR Green real-time RT-PCR in CSF and serum [3,4].
- *Rickettsia* spp. infections were detected using Hydrolysis probe RT-PCR in CSF [5,6]; Hydrolysis probe real time PCR and conventional PCR from buffy coat; and genetic sequencing.
- *R. typhi* and *Orientia tsutsugamushi* infections were detected using Hydrolysis probe real time PCR in CSF [5,6]; Hydrolysis probe real time PCR from buffy coat; and IgM and IgG assays from admission and follow-up serum (if there was a > 4-fold rise in antibody at follow-up) [7].
- *Leptospira* spp. infections were detected using hydrolysis probe real-time PCR in CSF [8]; culturing of blood clot on EMJH medium; microscopic agglutination tests at admission and follow-up (if there was a > 4-fold rise in antibody at follow-up)[9]; and hydrolysis probe real time RT-PCR from buffy coat [8].

The final etiology was determined based on the panel of diagnostic tests, including direct detection of pathogens in CSF or blood, IgM in CSF, seroconversion, or a 4-fold increase in antibody titer between the date of admission and follow-up serum samples. When more than 1 pathogen was present, direct tests were prioritized over indirect tests and presence in the CSF was prioritized over presence in the blood.

**SUPPLEMENTAL REFERENCES**

1. Dubot-Pérès A, Mayxay M, Phetsouvanh R, Lee SJ, Rattanavong S, Vongsouvath M, et al. Management of Central Nervous System Infections, Vientiane, Laos, 2003–2011. Emerg Infect Dis. 2019;25:898–910.

2. Leparc-Goffart I, Baragatti M, Temmam S, Tuiskunen A, Moureau G, Charrel R, et al. Development and validation of real-time one-step reverse transcription-PCR for the detection and typing of dengue viruses. J Clin Virol. 2009;45:61–6.

3. Moureau G, Temmam S, Gonzalez JP, Charrel RN, Grard G, de Lamballerie X. A real-time RT-PCR method for the universal detection and identification of flaviviruses. Vector Borne Zoonotic Dis. 2007;7:467–77.

4. Moureau G, Ninove L, Izri A, Cook S, De Lamballerie X, Charrel RN. Flavivirus RNA in phlebotomine sandflies. Vector Borne Zoonotic Dis. 2010;10:195–7.

5. Jiang J, Chan T-C, Temenak JJ, Dasch GA, Ching W-M, Richards AL. Development of a quantitative real-time polymerase chain reaction assay specific for Orientia tsutsugamushi. Am J Trop Med Hyg. 2004;70:351–6.

6. Jiang J, Stromdahl EY, Richards AL. Detection of Rickettsia parkeri and Candidatus Rickettsia andeanae in Amblyomma maculatum Gulf Coast Ticks Collected from Humans in the United States. Vector-Borne and Zoonotic Diseases. 2011;12:175–82.

7. Phetsouvanh R, Blacksell SD, Jenjaroen K, Day NPJ, Newton PN. Comparison of Indirect Immunofluorescence Assays for Diagnosis of Scrub Typhus and Murine Typhus Using Venous Blood and Finger Prick Filter Paper Blood Spots. The American Journal of Tropical Medicine and Hygiene. 2009;80:837–40.

8. Thaipadunpanit J, Chierakul W, Wuthiekanun V, Limmathurotsakul D, Amornchai P, Boonslip S, et al. Diagnostic Accuracy of Real-Time PCR Assays Targeting 16S rRNA and lipl32 Genes for Human Leptospirosis in Thailand: A Case-Control Study. PLOS ONE. 2011;6:e16236.

9. Cole JR, Sulzer CR, Pursell AR. Improved Microtechnique for the Leptospiral Microscopic Agglutination Test1. Appl Microbiol. 1973;25:976–80.
