# Supplementary material for: Spatial epidemiology of Japanese encephalitis virus and other infections of the central nervous system infections in Lao PDR (2003–2011): A retrospective analysis
Source: PLoS Negl Trop Dis. 2020 May 26;14(5):e0008333. doi: 10.1371/journal.pntd.0008333 (PMC7274481; doi:10.1371/journal.pntd.0008333)
Supplement: S2 Text — (DOCX) [file pntd.0008333.s002.docx]

**S2 text: Spatial point patterns**

The spatial distribution of villages from which study patients originated were indicated through maps of village locations, standard distance deviations (SDDs), and standard deviational ellipses (SDEs). Both SDDs and SDEs provide a visual representation of the central tendency and spread of points across a landscape [1,2]. SDEs also indicate potential anisotropy.

The SDD gives an indication of how points deviate from the mean center. The formula for the SDD is:

$$SDD=\sqrt{\frac{\sum_{i=1}^{n} {(x_{i}-X_{MC})}^{2}+ \sum_{i=1}^{n} {(y_{i}-Y_{MC})}^{2}}{n}}$$

where *x_i_* and *y_i_* are geographic references for point *i*;

{*X_MC_*, *Y_MC_*} is the geometric mean center (MC) for the features.

The SDE differs from the SDD in that the X- and Y-axes are calculated separately and the orientation is not necessarily horizontal/vertical. The Y-axis is rotated clockwise until the sum of the squares of the distances between points (village locations) and axes are minimized. The angle is defined as:

$$\theta=arctan\left\{ \frac{\left[ \sum_{i=1}^{n} \left( x_{i}-X_{MC} \right)^{2}-\sum_{i=1}^{n} \left( y_{i}-Y_{MC} \right)^{2} \right]+\sqrt{\left[ \left\{ \left( \sum_{i=1}^{n} \left( x_{i}-X_{MC} \right)^{2}-\sum_{i=1}^{n} \left( y_{i}-Y_{MC} \right)^{2} \right) \right\}^{2}+4\left\{ \sum_{i=1}^{n} \left( x_{i}-X_{MC} \right)\left( y_{i}-Y_{MC} \right)^{2} \right\} \right]}}{2\sum_{i=1}^{n} \left( x_{i}-X_{MC} \right)\left( y_{i}-Y_{MC} \right)} \right\}$$

The standard deviation is then calculated along both the shifted X- and Y-axes:

$$s_{X}= \sqrt{\frac{\sum_{i=1}^{n} \left[ \left( x_{i}-X_{MC} \right)\cos\theta-\left( y_{i}-Y_{MC} \right)\sin\theta\right]}{n}}$$

$$s_{Y}=\sqrt{\frac{\sum_{i=1}^{n} \left[ \left( x_{i}-X_{MC} \right)\sin\theta+\left( y_{i}-Y_{MC} \right)\cos\theta\right]}{n}}$$

.

The output of these statistics is traditionally mapped as an ellipse; with 1, 2, or 3 standard deviations (roughly corresponding to 63, 98, or 99 % of all geographic points, respectively). Spatial point patterns that are isotropic will result in an SDE that is equal to the standard distance deviation (SDD), resulting in a circular map layer rather than an ellipse.

Both the SDD and SDE can be weighted (for example, if multiple cases come from a single location).

**SUPPLEMENTAL REFERENCES**

1. Mitchell A. The ESRI Guide to GIS Analysis. ESRI Press; 2005.

2. Levine N. Spatial Distribution. CrimeStat III: a spatial statistics program for the analysis of crime incident locations (version 30) [Internet]. Houston, TX; Washington DC: Ned Levine & Associates; National Institute of Justice; 2004. Available from: https://www.nij.gov/topics/technology/maps/documents/crimestat-files/CrimeStat%20IV%20Chapter%204.pdf
