# Supplementary material for: Spatial epidemiology of Japanese encephalitis virus and other infections of the central nervous system infections in Lao PDR (2003–2011): A retrospective analysis
Source: PLoS Negl Trop Dis. 2020 May 26;14(5):e0008333. doi: 10.1371/journal.pntd.0008333 (PMC7274481; doi:10.1371/journal.pntd.0008333)
Supplement: S3 Text — (DOCX) [file pntd.0008333.s003.docx]

**S3 text: Environmental Indices (EIs)**

The photosynthetic components of vegetation (i.e. chlorophyll) absorb visible light, especially in the Red and Blue wavelengths. Conversely, most infrared radiation is reflected by healthy vegetation. The contrast between Red and near-infrared (NIR) responses therefore provides an estimate of healthy vegetation.

One common measure of landscape vegetation is the normalized difference vegetation index (NDVI [1]) which is frequently defined as:

$$NDVI= \frac{NIR-Red}{NIR+Red}$$

.

This simple measurement is sensitive to atmospheric effects and dense canopy structure [2]. While NIR can pass through multiple layers of canopy structure, Red typically cannot. In areas with high vegetation density NDVI quickly becomes saturated. An improved metric has been developed to account for these problems, referred to as the enhanced vegetation index (EVI [3]). This metric uses the difference between Red and Blue reflectances as an estimator of atmospheric influence level on the vegetation index. EVI is commonly specified as:

$$EVI=G\frac{NIR-Red}{NIR+C_{1}Red-C_{2}Blue+L}$$

; where *L* is the canopy background adjustment;

*C*_1_ and *C*_2_ are coefficients of an aerosol resistance term;

and *G* is a scaling factor.

A variety of similar indices have been proposed to measure water content, either within vegetation (i.e. measuring drought conditions or identifying areas that have been burned) or as surface water. In general, indices that use a combination of NIR and shortwave infrared responses (SWIR) have been proposed to measure within-vegetation water content whereas those that use a combination of visible spectral regions (VIS) and SWIR are usually proposed for identifying water bodies.

Almost all include a SWIR component because infrared in these wavelengths are well- absorbed by water (see [4], for example). Following Boschetti et al [5] we use the following normalized flooding index (NFI):

$$NFI=\frac{Red-SWIR2}{Red+SWIR2}$$

; where SWIR2 is shortwave infrared radiation 2 (~ 1640nm).

**SUPPLEMENTAL REFERENCES**

1. Rouse J, Hass R, Deering D, Sehell J. Monitoring the vernal advancement and retrogradation (Green wave effect) of natural vegetation [Internet]. 1974 p. 8. Report No.: E74-10676, NASA-CR-139243, PR-7. Available from: https://ntrs.nasa.gov/archive/nasa/casi.ntrs.nasa.gov/19740022555.pdf

2. Huete AR. A soil-adjusted vegetation index (SAVI). Remote Sensing of Environment. 1988;25:295–309.

3. Huete A, Didan K, Miura T, Rodriguez E, Gao X, Ferreira L. Overview of the radiometric and biophysical performance of the MODIS vegetation indices. Remote Sensing of Environment. 2002;83:195–213.

4. Hale GM, Querry MR. Optical Constants of Water in the 200-nm to 200-μm Wavelength Region. Appl Opt, AO. 1973;12:555–63.

5. Boschetti M, Nutini F, Manfron G, Brivio PA, Nelson A. Comparative Analysis of Normalised Difference Spectral Indices Derived from MODIS for Detecting Surface Water in Flooded Rice Cropping Systems. Schumann GJ-P, editor. PLoS ONE. 2014;9:e88741.
