# Supplementary material for: Spatial epidemiology of Japanese encephalitis virus and other infections of the central nervous system infections in Lao PDR (2003–2011): A retrospective analysis
Source: PLoS Negl Trop Dis. 2020 May 26;14(5):e0008333. doi: 10.1371/journal.pntd.0008333 (PMC7274481; doi:10.1371/journal.pntd.0008333)
Supplement: S4 Text — (DOCX) [file pntd.0008333.s004.docx]

**S4 text: Statistical model selection**

Formal multivariable analysis was conducted on both the village- and individual-level datasets. Small case numbers for mono-infections limited our multivariable analyses to an analysis of study patient home villages and comparison villages as well as the most commonly diagnosed infection: JEV. The village-level data were coded as a “1” or “0” based on whether or not the village was home to an study patient; and whether or not the village was home to an study patient diagnosed with JEV. The individual-level dataset was likewise coded with a “1” or “0” based on whether or not the individual was diagnosed with a JEV infection (all patients in the individual-level data had an LP).

We began with an exploratory multivariable analysis using generalized additive models (GAMs) with a binomial distribution (logistic GAMs). The use of GAMs allowed us to explore the potentially non-linear shape of the association between continuous environmental predictors (NDVI, EVI, and NFI) and the outcome variables and informed our final model selection and variable specification.

Our first GAMs included both NDVI and EVI, which are considered complimentary to each other [1]. The models showed a high degree of concurvity between these two covariates, almost no added benefit (from model fit statistics), and no detectable effect of the NDVI covariate. In subsequent models we therefore retained only EVI as a measure of vegetation.

Village-level GAMs began with village geographic (elevation, distance to nearest major road) and demographic (village population size) covariates. A second model was then specified including the environmental covariates at the 2km buffer size around each village. Subsequent models tested larger buffer sizes (5km and 10km), investigating overall model fit using the Akaike Information Criterion (AIC) and the explained deviance. The smoothed functions were chosen using restricted maximum likelihood (REML).

The village-level GAM for all study patients (that is, all patients who had an LP regardless of diagnosis) showed statistically significant contributions from NFI, EVI, village population, distance to the nearest major road, and elevation. The effects of NFI, EVI, and village population were positive while the effects for distance to the nearest major road and elevation were negative. The effects for NFI, EVI, village population, and elevation all appeared curvilinear. The village level model for JEV villages (villages from which LP patients who were diagnosed with JEV came) indicated that only village population was a significant predictor, with a curvilinear effect.

Individual-level models began with village- (village population, elevation, distance to the nearest major road) and individual- (age, gender, admission quarter and year) level variables. Environmental variables were first added at the 2km buffer size and for the year prior to admission. Subsequent models tested larger buffer sizes until the AIC was minimized. The 10km buffer appeared to provide the best model fit. The temporal resolution was then varied at the 10km buffer size, beginning with 1 year mean prior to admission, then 2 months mean prior to admission, and finally within the same month as admission. The best fitting model appeared to be the 10km buffer and measures from the same month as admission.

Both NFI and EVI show seasonal variations and calendar month is a strong predictor of NFI. In order to account for concurvity in the GAMs and collinearity in the subsequent logistic generalized linear models, we transformed the environmental variables to quartiles for subsequent models. This transformation allows for easy interpretation of covariate effects, allows for non-linear associations between the covariate and the outcome of interest, and allows the model to simultaneously address both the seasonality in cases (especially JEV) and the apparent associations with surface water (NFI).

All other continuous variables were centered on their means and standardized by their standard deviations.

The final village-level model was a logistic regression and the final individual-level model was a mixed effects logistic regression, with a random intercept for village, both using these transformed and standardized variables.

**SUPPLEMENTAL REFERENCES**

1. Huete A, Didan K, Miura T, Rodriguez E, Gao X, Ferreira L. Overview of the radiometric and biophysical performance of the MODIS vegetation indices. Remote Sensing of Environment. 2002;83:195–213.
