# Supplementary material for: Spatial epidemiology of Japanese encephalitis virus and other infections of the central nervous system infections in Lao PDR (2003–2011): A retrospective analysis
Source: PLoS Negl Trop Dis. 2020 May 26;14(5):e0008333. doi: 10.1371/journal.pntd.0008333 (PMC7274481; doi:10.1371/journal.pntd.0008333)

**S1 fig**: Diagram of data processing and aggregation. Four different data sources are used (indicated by boxes with dashed lines). Two main datasets are created from the combined sources (indicated by boxes shaded in grey): an individual-level dataset (one row per patient) and a village-level dataset (one row per village). NDVI indicates the normalized differential vegetation index, EVI indicates the enhanced vegetation index, and NFI indicates the normalized flooding index.


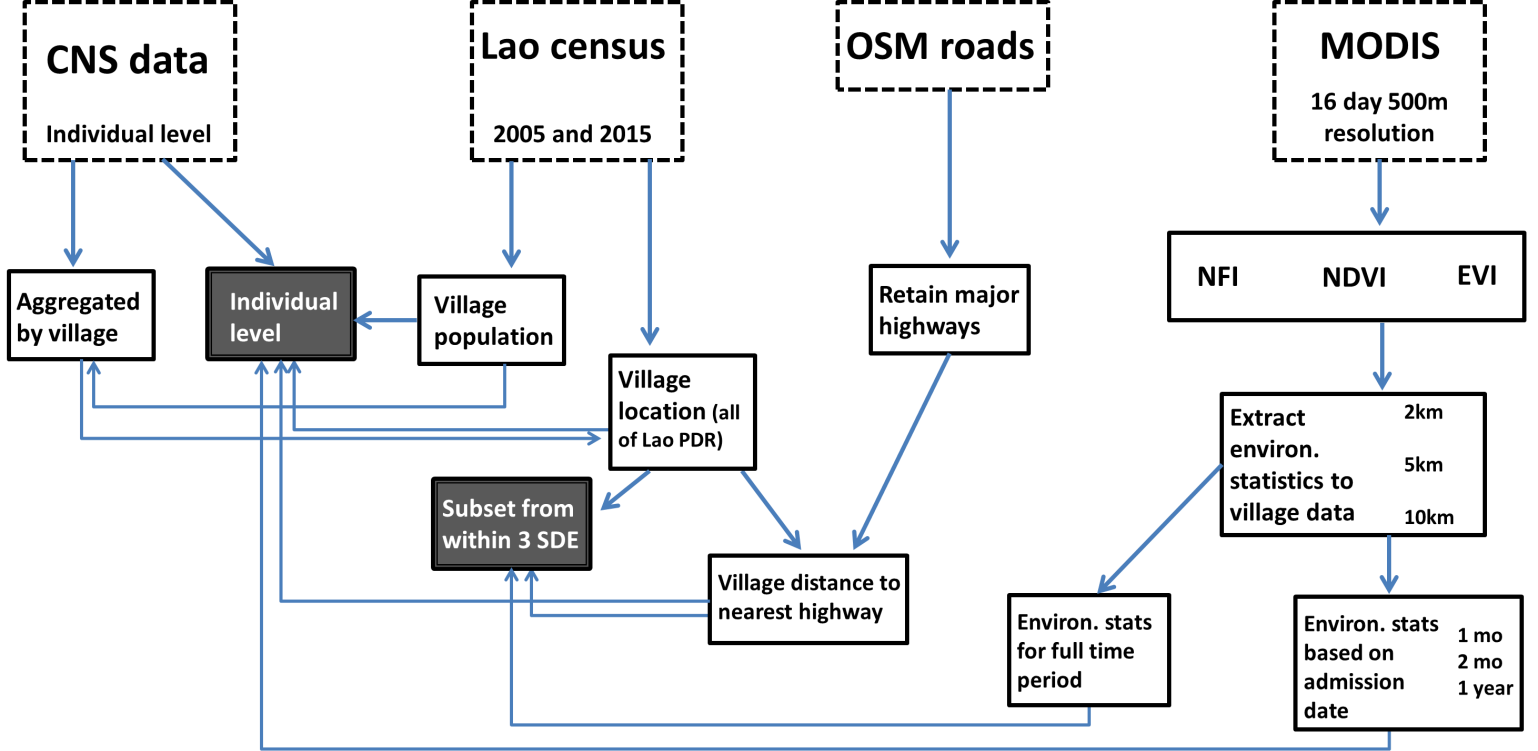

Supplement: S1 Fig — Four different data sources are used (indicated by boxes with dashed lines). Two main datasets are created from the combined sources (indicated by boxes shaded in grey): an individual-level dataset (one row per patient) and a village-level dataset (one row per village). NDVI indicates the normalized differential vegetation index, EVI indicates the enhanced vegetation index, and NFI indicates the normalized flooding index. (DOCX) [file pntd.0008333.s005.docx]
