# Supplementary material for: Spatial epidemiology of Japanese encephalitis virus and other infections of the central nervous system infections in Lao PDR (2003–2011): A retrospective analysis
Source: PLoS Negl Trop Dis. 2020 May 26;14(5):e0008333. doi: 10.1371/journal.pntd.0008333 (PMC7274481; doi:10.1371/journal.pntd.0008333)

**S2 fig**: Subset of villages selected for village level analysis. A standard deviational ellipse ((SDE) with 3 standard deviations) was drawn around the home villages of all LP patients. All villages within that SDE were selected for the village level analysis.


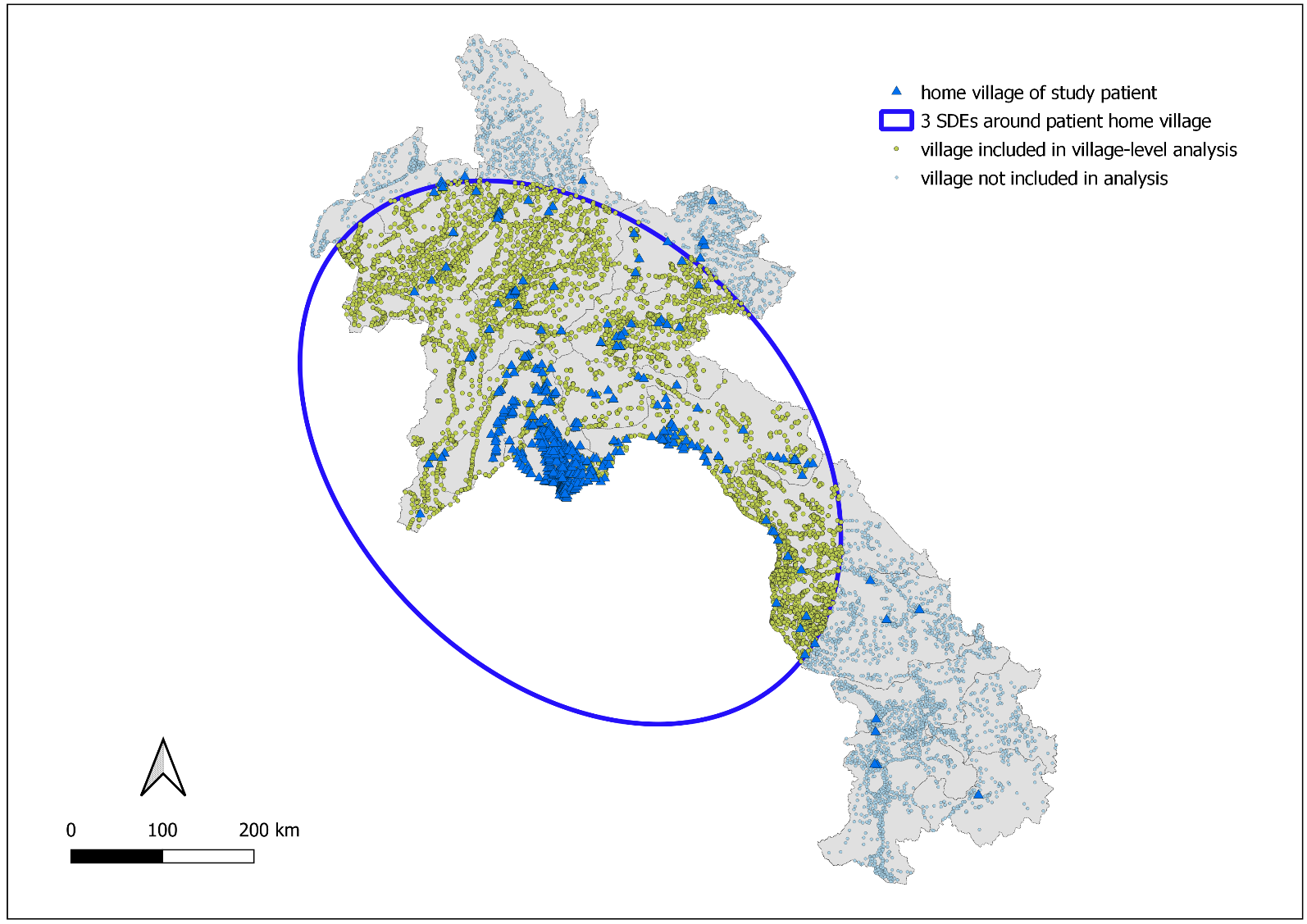

Supplement: S2 Fig — A standard deviational ellipse ((SDE) with 3 standard deviations) was drawn around the home villages of all LP patients. All villages within that SDE were selected for the village level analysis. (DOCX) [file pntd.0008333.s006.docx]
