# Supplementary material for: Spatial epidemiology of Japanese encephalitis virus and other infections of the central nervous system infections in Lao PDR (2003–2011): A retrospective analysis
Source: PLoS Negl Trop Dis. 2020 May 26;14(5):e0008333. doi: 10.1371/journal.pntd.0008333 (PMC7274481; doi:10.1371/journal.pntd.0008333)
Supplement: S3 Fig — Major roads (dark black lines) downloaded from OpenStreetMaps (2017) for use in calculating the Euclidian distance from each village to the nearest major road. Roads included “primary”, “secondary”, and all major connecting roads. (DOCX) [file pntd.0008333.s007.docx]

**S3 fig**: Major roads (dark black lines) downloaded from OpenStreetMaps (2017) for use in calculating the Euclidian distance from each village to the nearest major road. Roads included “primary”, “secondary”, and all major connecting roads.

**
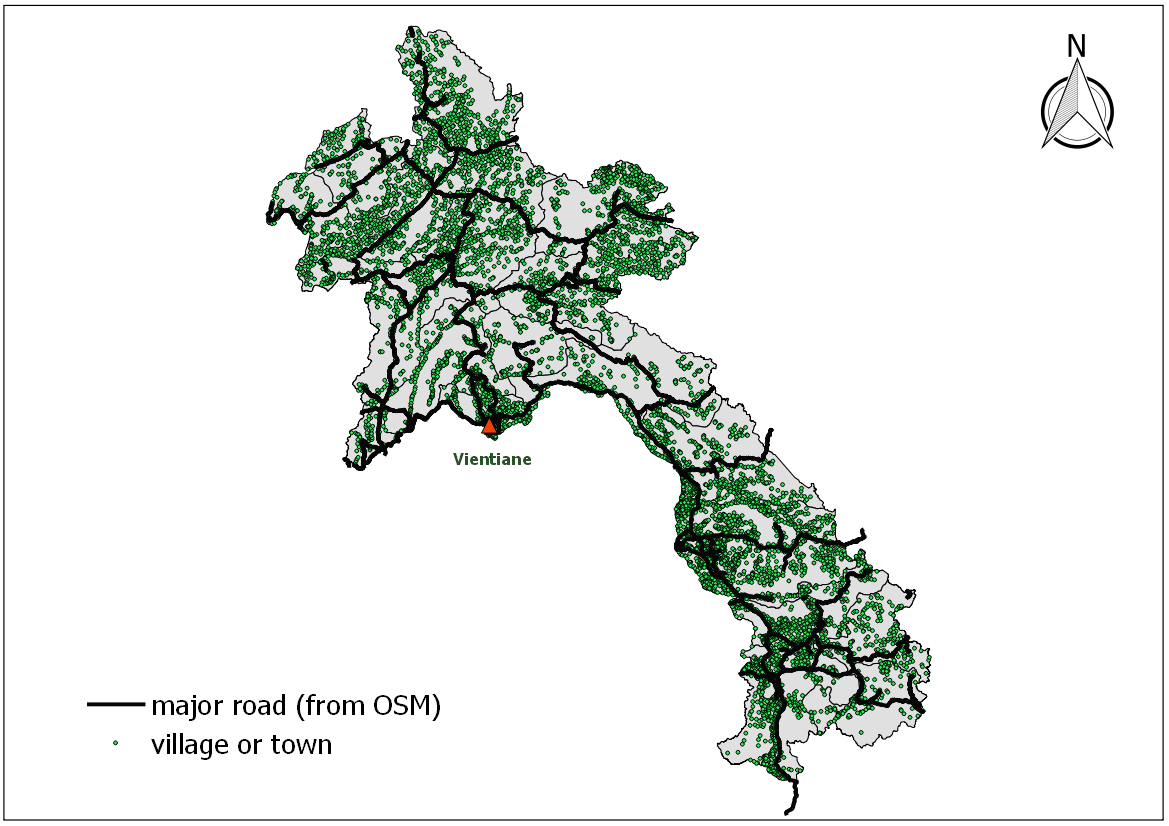
**
