# Supplementary figures and images for: Spatial epidemiology of Japanese encephalitis virus and other infections of the central nervous system infections in Lao PDR (2003–2011): A retrospective analysis
Source: PLoS Negl Trop Dis. 2020 May 26;14(5):e0008333. doi: 10.1371/journal.pntd.0008333 (PMC7274481; doi:10.1371/journal.pntd.0008333)

**S4 fig:** Mean village population (and 95% CI) by diagnosis


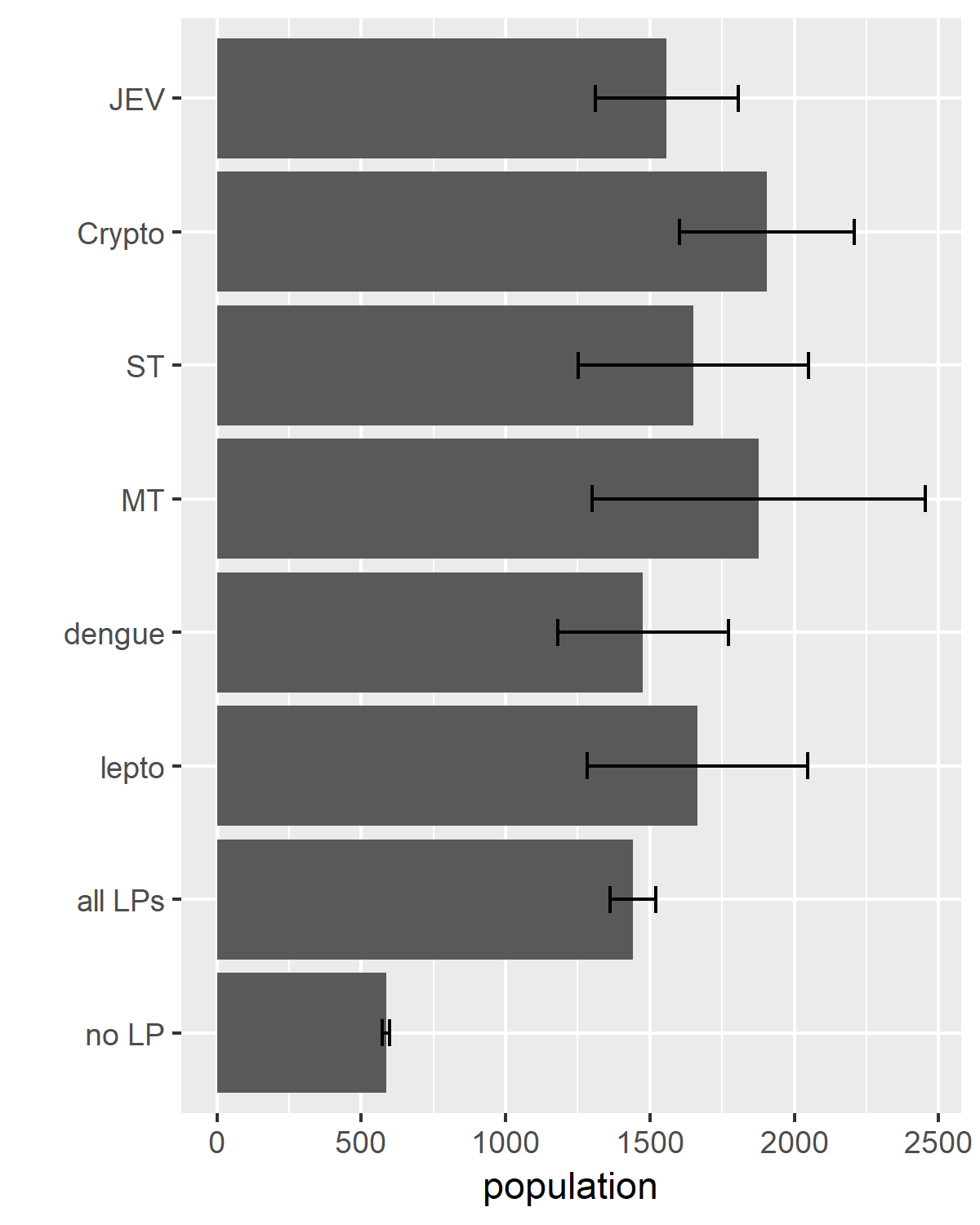

Supplement: S4 Fig — (DOCX) [file pntd.0008333.s008.docx]

**S5 fig:** Mean village elevation (and 95% CI) by LP diagnosis

**
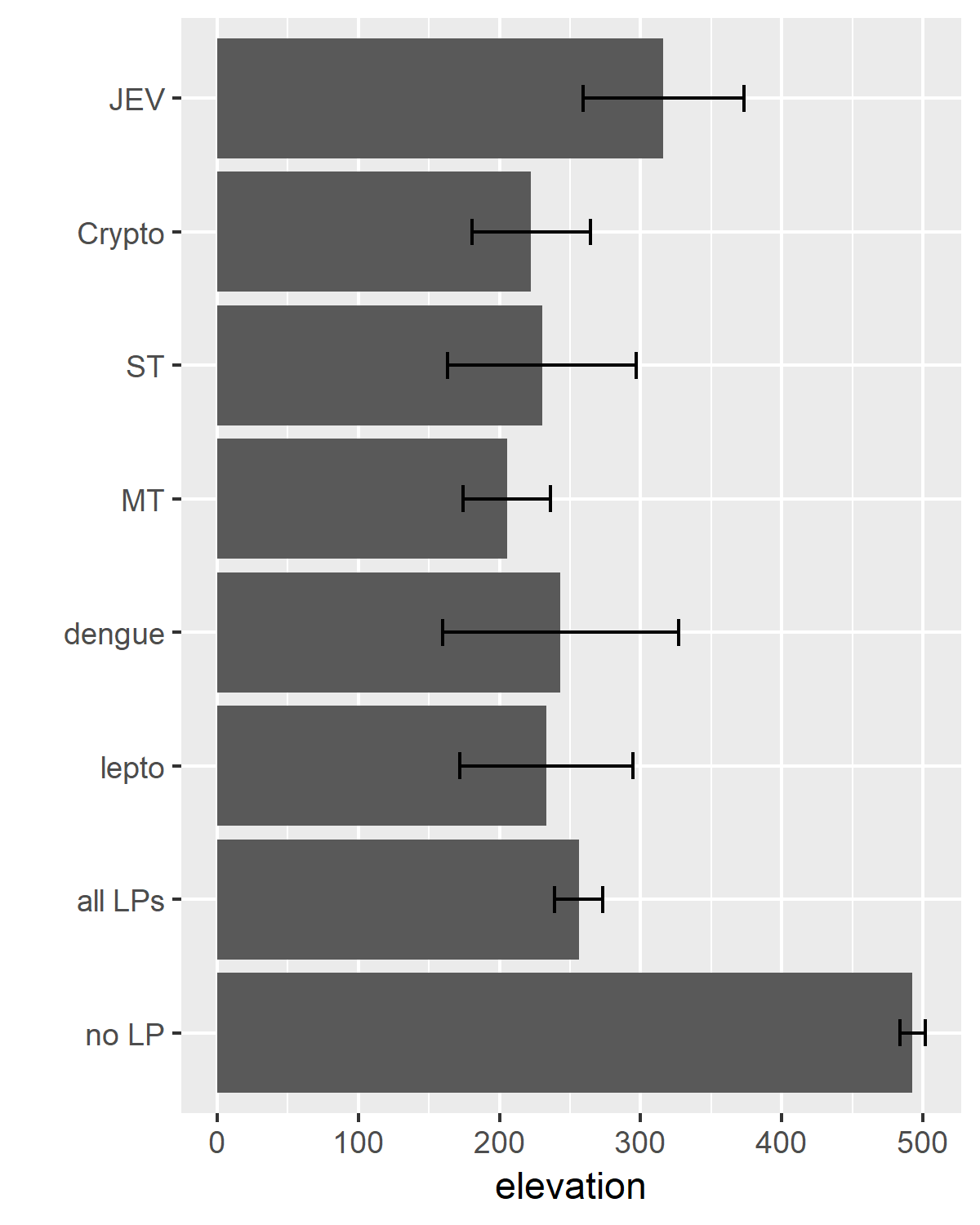
**

Supplement: S5 Fig — (DOCX) [file pntd.0008333.s009.docx]
